# Preparation and Characterization of Multilayer NiTi Coatings by a Thermal Plasma Process

**DOI:** 10.3390/ma17030694

**Published:** 2024-02-01

**Authors:** Sneha Samal, Jakub Zeman, Stanislav Habr, Oliva Pacherová, Jaromír Kopeček, Petr Šittner

**Affiliations:** FZU-Institute of Physics of Czech Academy of Science, Na Slovance 1999/2, 18200 Prague, Czech Republic; zemanja@fzu.cz (J.Z.); habr@fzu.cz (S.H.); pacher@fzu.cz (O.P.); kopecek@fzu.cz (J.K.); sittner@fzu.cz (P.Š.)

**Keywords:** thick NiTi coating, thermal plasma process, deposition, characterization

## Abstract

The deposition of multilayer coating of NiTi is carried out by a thermal plasma spraying process on a stainless steel substrate. The deposition of melted NiTi particles creates an adhesion layer on the substrate with the subsequent formation of multilayer coating with a certain thickness. Six layers of coating are created to achieve a certain thickness in terms of the sprayed sample. This paper aims to investigate multilayer NiTi coatings created through a thermal plasma process. The key variable feed rate was considered, as well as its effect on the microstructure characteristics. The shape memory effect associated with the coating properties was analyzed in detail. The variable feed rate was considered one of the most important parameters in the thermal plasma spraying process due to its ability to control the quality and compactness of the coating structure. The coatings were characterized by examining their microstructure, thermal, chemical, and microhardness. The indent marks were made/realized along the cross-section surface for the analysis of crack propagation resistance and wear properties. The coating’s surface did not display segmentation crack lines. Nevertheless, the cross-sectional surfaces showed evidence of crack lines. There were eutectic zones of the interlamellar structure observed in the structure of the coating. The plasma-sprayed samples from thermo-mechanical analysis of the hysteresis curve provide strong confirmation of the shape memory effect.

## 1. Introduction

To obtain high thrust and efficient performance in aerospace alloys, NiTi coatings have been implemented on surface alloys and metal surfaces; for example, [1,2,3,4] studied the application of ceramic barrier coatings for metals and alloys, which can be used in aggressive environments under temperatures close to their melting point. Metals have excellent mechanical properties and corrosion resistance, and are, therefore, useful in industrial applications [5,6]. However, despite technological advancements in various sectors, it is very challenging to meet the demand with stainless steel performance. NiTi alloy has gained popularity due to its superelasticity, shape memory, and wear resistance; it can be considered to have good potential as a coating for effectively improving surface properties [7]. The implementation of NiTi powder as a coating layer can effectively improve a material’s performance, thus extending its durability over its lifetime as service part in the field of wear behavior [8,9,10]. NiTi coatings are implemented as functional protective layers in cavitation and to prevent erosion behavior by improving the surface properties [11,12,13]. However, current research mostly focuses on the application of NiTi as bulk material in various aspects of biomedical industries, such as stents and guide wire [14]. Researchers are mostly focused on fatigue behavior, corrosion resistance, and improving the lifetime of NiTi as the primary material to understand superelasticity and martensitic phase transformation processes [15,16]. However, in the field of additive manufacturing, a limited number of researchers [17,18,19,20,21,22] have studied alternative routes, such as the powder metallurgy route of additive manufacturing, laser melting, and direct energy deposition processes for bulk NiTi materials. The process of powder metallurgy results in the disintegration of the composition from the stoichiometric composition of the 50Ni:50Ti alloy, which could lead to deviations in the behavior of shape memory alloys [23,24]. Proper material design plays an important role in the advancement of the engineering field. Thermal plasma spray technology opens up the possibility of spraying multiple layers in various patterns to enhance a material’s surface properties in various potential application areas [25,26,27].

The purpose of coating stainless steel is that it provides insights into multilayer coatings designed and fabricated using thermal plasma technology. The powder feeding rate varied for each multilayer coating structure on the substrate of stainless steel with fixed experimental parameters using the plasma spraying process. Recently, a thermal plasma process has emerged in the processing of powder coatings on the surfaces of materials that could enhance the performance of the surface properties. A lack of understanding about the compactness of the coating structure arises from the effect of thermal plasma spraying parameters, as identified in our previous studies [28,29,30]. In this work, two samples were chosen with variable feed rates to create multilayer coating with varying quality and compactness. The multilayer coating was fabricated on the surface of a stainless steel substrate. The lamellar interface between various layers of the coating was examined in terms of dense porosity, and segmentation crack lines. The difference in the coefficient of thermal expansion (CTE) between the substrate and coating layer may influence interfacial cracks during the spraying process [31,32]. The quality of the coating layers, which determines the compactness and porosity generated during the thermal plasma process, may have a strong influence on the performance of the material [33]. Therefore, in this study, multilayer coating was prepared by varying the powder feed rates on the stainless steel substrate. The adhesion between the substrate and the coating and that between the segmented layers was studied and analyzed in consideration of the overall performance of the multilayer coating structure.

This work provides good insight into the preparation of NiTi layers through the use of plasma spraying, both in terms of preparation methods and characterization. This paper aims to investigate multilayer NiTi coatings fabricated through the use of the thermal plasma process. The primary variable feed rate was taken into consideration, along with its impact on the microstructure characteristics. The shape memory effect associated with the coating properties was analyzed in detail. The NiTi coating could be used as a protective layer for the material, enhancing SE and SMA behavior against wear, erosion, and corrosion.

## 2. Materials and Methods

### 2.1. Materials

**Figure 1:**
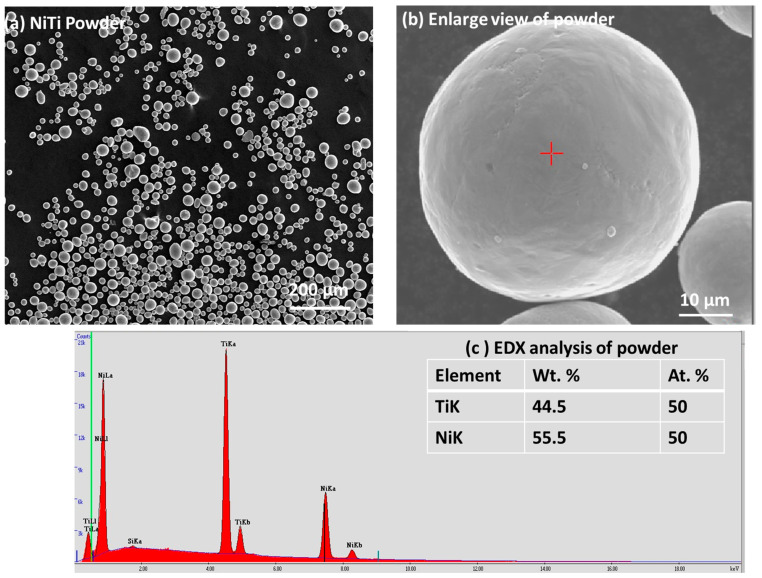
(**a**) Particles of NiTi considered for thermal plasma coating; (**b**) one-particle view with red cross showing EDX location; (**c**) EDX analysis of elements [7] (open access from MDPI).

The functional coating was deposited by using NiTi powder as feedstock raw material and stainless steel as the substrate. NiTi powders were purchased, rather by choosing the selective composition of each particle with a major composition of 50 at. % of Ni and Ti with a purity of 99.5% and particle sizes ranging from 20 to 63 μm, from American Elements, MERELEX Corporation, Los Angeles, CA, USA. There were other elements, such as Fe, Al, Cu, Si, Ca, C, O, and N, present in minor amounts (less than ≤ 0.01–0.005) [31]. Figure 1a,b displays the particle size and distribution of NiTi powder in a larger view, and one enlarged particle shows the surface features with a red cross mark, where Energy-Dispersive Analysis (EDX) was performed. Figure 1c shows the EDX peaks with the composition of Ni and Ti within the inset table. The dimension of 60 × 20 × 3 mm were considered for the stainless steel.

### 2.2. Experimental Methods

**Figure 2:**
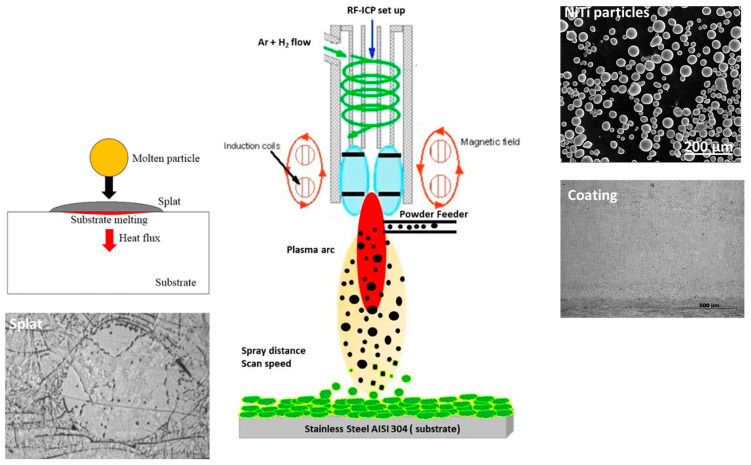
Schematic representation of the spraying process showing powder deposition on the stainless steel substrate [34] (open access from MDPI).

The samples were fabricated by the facility of thermal plasma spraying (RF-ICP) from IPP, Czech Republic. The coatings were deposited by using natural argon gas with a mixture of hydrogen. Argon was the chosen carrier gas, which was used as a powder feeder to the plasma system. The spraying was performed at a plasma power of 9 kW for both samples with lamellar layers of deposition that developed along the thickness at two different feed rates. A spraying speed of 1 mm/s was chosen for both samples, with preheating for 60 s for each spraying pass. Both samples show six lamellar layers in coating thickness, with the same amount of powder spray and constant power. The variable parameter was the feed rate of powder used in the thermal plasma spraying method. This difference affects the effect, porosity, and interlamellar cracks within the segmented layers, determining the structure of the coating. Figure 2 shows the thermal plasma deposition process. The particles melt along the path of the thermal plasma source, leading to particles impacting the surface. During impact, multiple splats are formed, forming layers of coating. Figure 2 shows the RF-ICP plasma process for coating deposition with particles and the splat, showing the formation of coating layers. Table 1 represents the spraying parameters for the preparation of two selected samples. Table 1 represents the experimental parameters for the preparation of samples 1 and 2 with different feed rates of powder spraying. Argon was used as the plasma gas to create the plasma arc that induces the melting of particles and deposition of the coating layers. The substrate underwent preheating before the application of powders in a plasma arc. The spraying was performed for 60 s six times to maintain thick multilayered coating layers. A 15 s break was considered for the cooling process in between each spraying.

### 2.3. Plasma-Sprayed Sample Characterization

Various techniques have been used to investigate the physical, microstructural, thermal, and mechanical behavior of the coatings. The surface image and quality of the coating samples were investigated by examining the cross-sectional images of the coatings. Initially, the coatings were separated by cutting the coating layers without and with coating by using an electric discharge machine (FI240 SLP). The surface image was investigated using Tescan FERA 3 (Tescan, Brno, Czech Republic) scanning electron microscopes (SEM), the latter equipped with a field emission cathode. The analyses were performed using either secondary electron imaging for topography or backscattered electrons for chemical Z contrast. Energy-dispersive X-ray spectroscopy (EDS) and line analysis were carried out using the EDAX system (EDAX, Ametek Inc., Mahwah, NI, USA) with an Octane Super 60 mm^2^ detector to determine the homogeneity of the chemical composition. The voltage used for EDS analysis was 15 kV, which was used to ensure the visibility of the K line for Ni. The phases in the plasma-sprayed samples were revealed through the use of an X-ray diffractometer using the PANalytical X’Pert Pro diffractometer (Malvern, UK) with Kα Co radiation. The hardness of the samples was determined by indenting the cross-section surfaces. A load of 1.961 N was used for 10 s for each indent point. Five indents were used for the calculation of the average hardness of the samples. Differential scanning calorimetry (DSC) using the DSC 25 (TA Instruments, New Castle, DE, USA) instrument at a scanning rate of 5 K/min in a temperature range from −100 to +100 °C (powders) and from 0 to +150 °C (samples) was performed. For these tests, small samples were placed and measured in an Al crucible (approximately 13.3 mg). The samples were tested in a three-point bending test at a fixed load of 100 mN for the shape memory effect. The experiment was performed according to the ASTM E831 standard [35] by using a thermo-mechanical analyzer (TMA, Linseis, Germany). The samples were tested within the temperature range of −25 to +130 °C.

## 3. Results

The microstructure determines the quality of the coating that is evaluated from the cross-section of the image. The porosity and interlamellar cracks along the various coating layers on the surface of the substrate are determined by the quality of the coating. The elemental composition and line analysis along the coating layers have been analyzed. Various phases of NiTi that formed in the coating layers are derived from the phase diagram of Ni and Ti as a function of the temperature. The transformation temperature of the phases in relation to heating and cooling is investigated in the coating samples. The transformation temperature frame was chosen for the sample bending behavior at a constant load to induce shape memory behavior.

### 3.1. Microstructural Observation in Coating Layers

**Figure 3:**
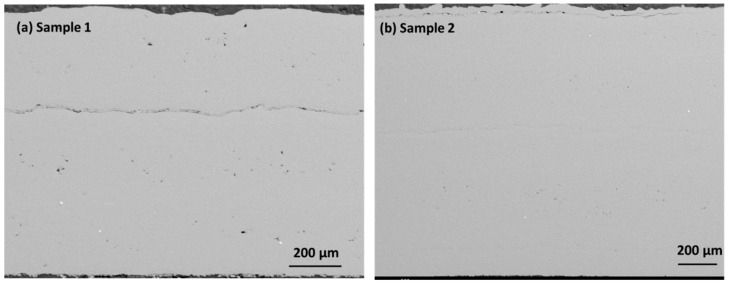
(**a**,**b**) Cross-section images along the cross-section for samples 1 and 2.

**Figure 4:**
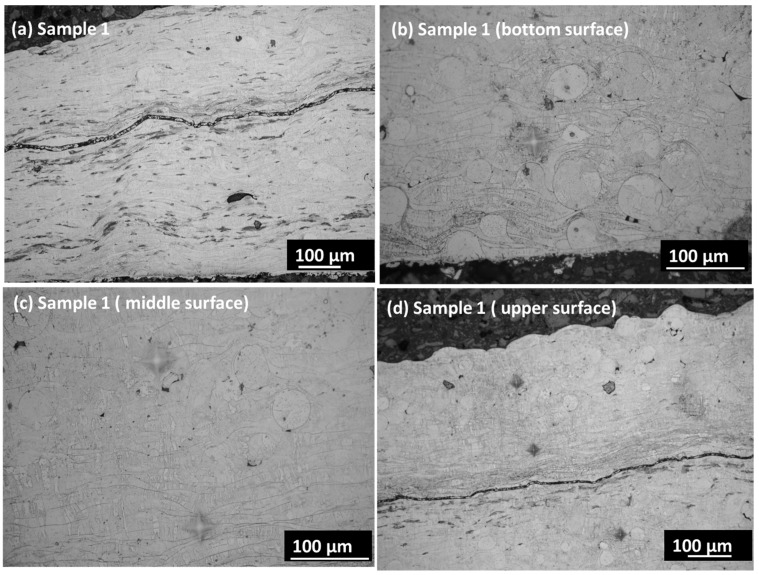
(**a**–**d**) Optical microscope image of sample 1; (**a**) overall surface; (**b**) bottom surface; (**c**) middle area; (**d**) upper surface area.

**Figure 5:**
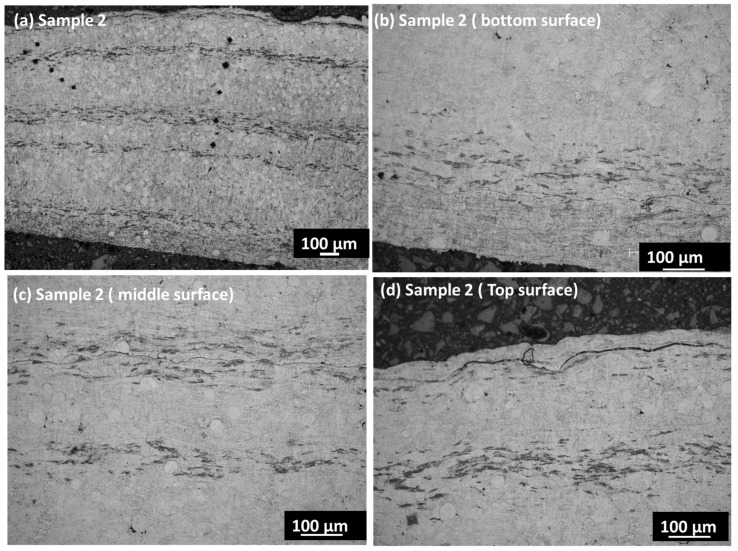
(**a**–**d**) Optical microscope image of sample 2; (**a**) overall surface; (**b**) bottom surface; (**c**) middle area; (**d**) upper surface area.

Figure 3a,b presents the cross-section images in the SE mode in both samples. Sample 1 shows minor porosity along the cross-section, which may have developed due to a lower feed rate. The low feeding rate may create a void within the coating layers due to entrapped air or oxygen within the layers. The porosity is prominently visible in sample 1 along the coating layers in the cross-section. When analyzing the coating’s cross-section, the segmentation crack lines are visible instead of on the coating’s surface. A segmentation crack line is observed in the middle of sample 1 cross-section surface. The crack line appears due to the weak interconnection of particles along the lamellar layer. However, this crack disappears with the better connection of particles at a higher feed rate. Sample 2 shows a crack line at the edge of the top surface that may have been generated during cooling. The various locations of the coating layer are revealed by etching the surface before using an optical microscope. Figure 4a,d displays the overall surface and the lower, middle, and top surface layers. The etching reveals the magnified surface image of the coating layers in various zones. The bottom surface shows larger splat droplets, which are arranged linearly along the thickness of the coating layer. However, the crack line is observed in the overall sample, which corresponds to the location within the upper part of the sample. This may arise due to the inter-surface separation between two adjacent layers due to the lower feed rate. The middle layers show dense, compact layers with minor porosity, which arises from the interconnected neighborhoods of the splat particles. The middle layer shows the columnar lines along the deposition height of the coating layers. Some interfacial cracks are found in the coatings, which may attributed to stress mismatch between the layers. The interfacial cracks arise from oxidized impurities within the layers that arise from the porosity regions. Figure 5a,d displays the overall surface and the bottom, middle, and top surfaces of the coating layers. There are compact layers of coating observed in the bottom layer, although the homogenized, compact microstructure also shows a continuation in the middle layer. There is a very thin crack line observed in the middle layers; however, the overall sample shows good integrity in terms of the coating layers. The segmented crack line shifted to the edge of the upper surface, which may arise from the cooling difference in sample 2. However, sample 2 is more compact and denser within the microstructure, with good layers in the coating surface that may have been achieved from the 4.2 g/min feed rate with very minor porosity.

### 3.2. Elemental Analysis within Coating Layers Using Image and Line Analysis

**Figure 6:**
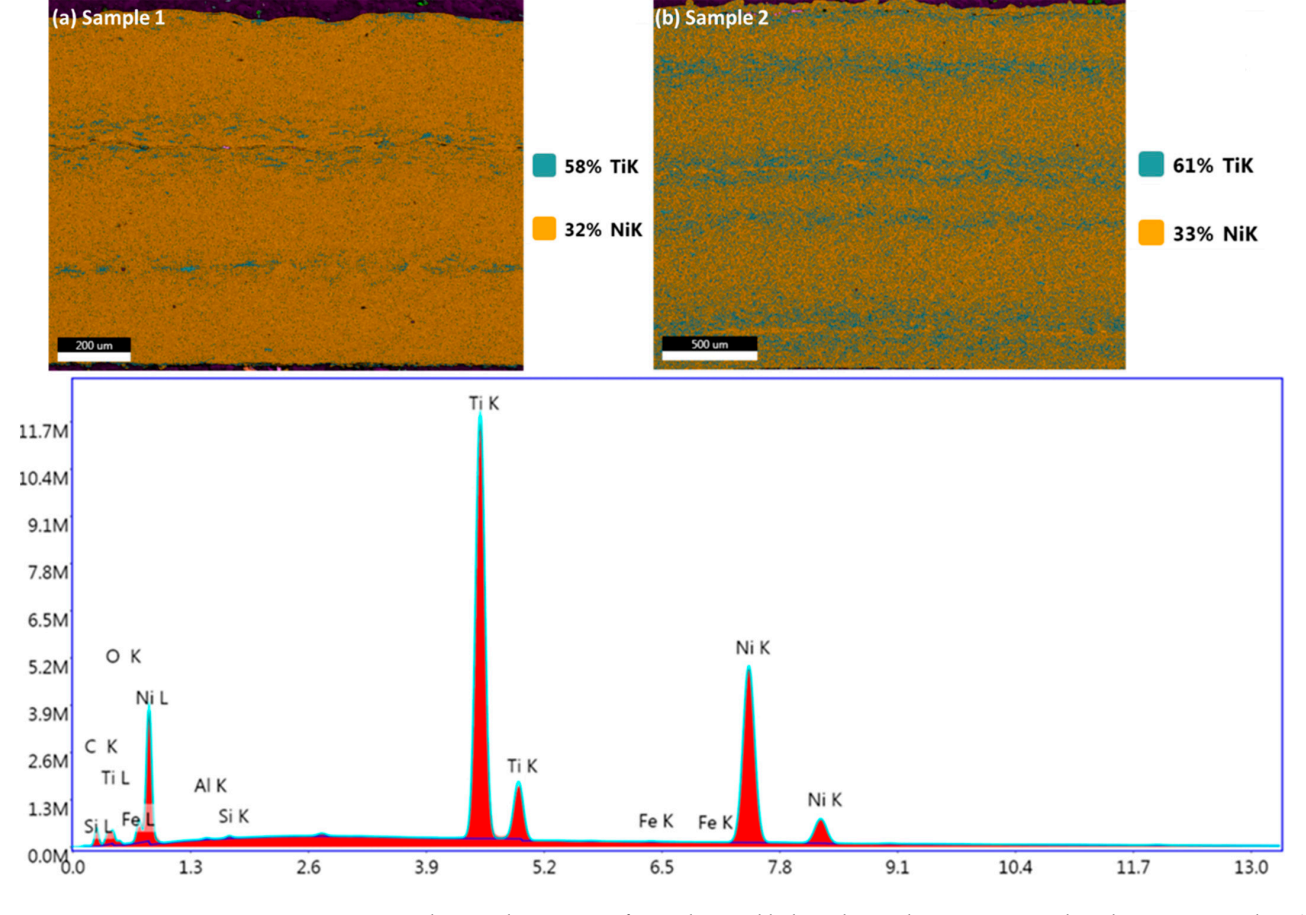
Elemental mapping of sample 1 and below shows the compositional analysis in Wt. and at.%.

**Figure 7:**
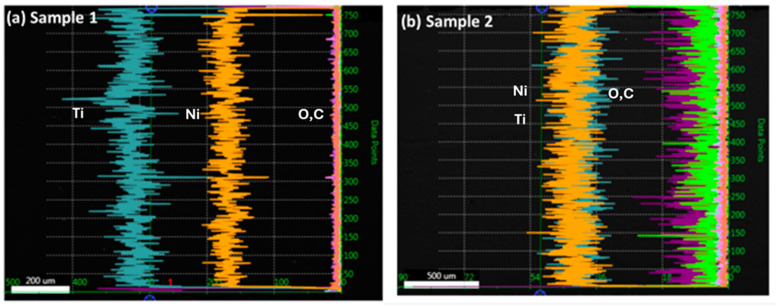
Line analysis in samples 1 and 2 shows the presence of a shift in the composition of Ni, Ti, C, and O content.

The presence of various elements within the coating layers is investigated by using image and line analysis using the SEM technique (Figure 6). The elements of Ni, Ti, and others are present in samples 1 and 2 when examining the EDX peaks. There are 5% and 2% C K in samples 1 and 2, respectively. Sample 1 displays 58% TiK, while sample 2 displays 61% TiK. In both samples 1 and 2, NiK is nearly equal. Sample 1 has more contaminants than sample 2, such as higher C concentration. Other elements, such as O, Al, Fe, and Si, are very minor. The line analyses for major elements such as Ni and Ti and minor elements such as C and O were performed for samples 1 and 2 (Figure 7). It has been observed that the Ni content is lower than the Ti content in sample 1, and it remains constant along the coating layers, with the minimum content of C and O. Nonetheless, sample 2 shows that, with a minimum content of C and O, Ni and Ti contents overlap in the same quantity over the coating layers, as shown in Figure 7a,b. Because both samples have different scales, the signal displays different ratios.

### 3.3. Phase Identification in the Coating Structure

**Figure 8:**
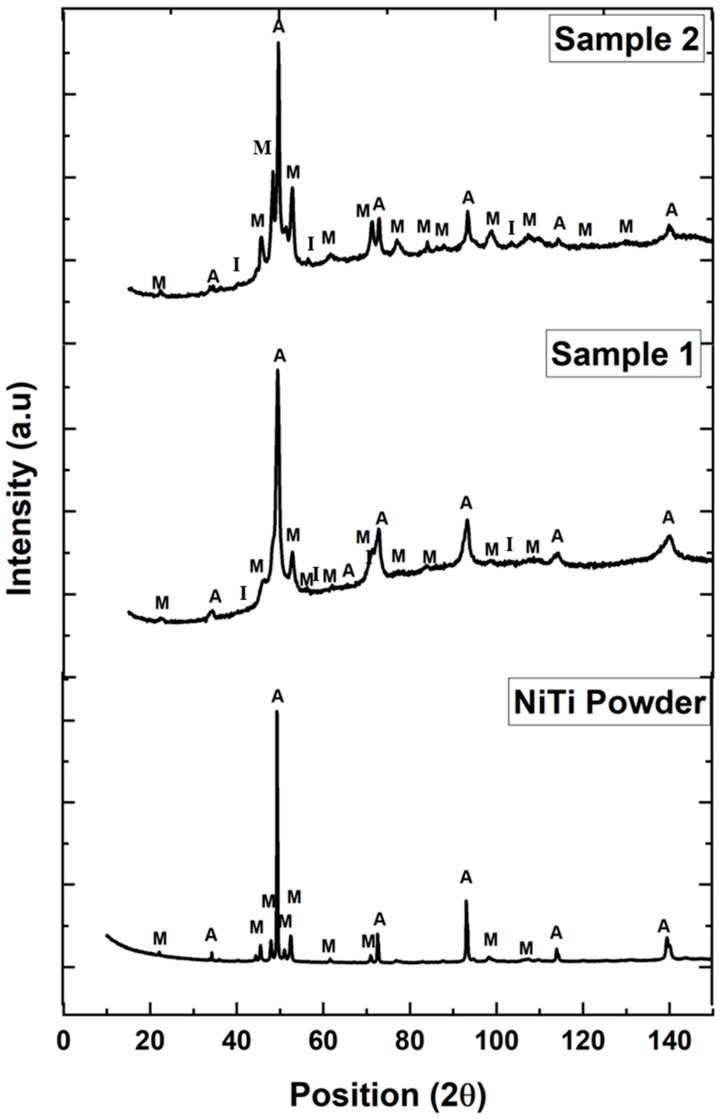
Phases of A: austenite (B2); M: martensite (B19′); and I: intermetallic (NiTi_2_) present in NiTi powder and plasma-sprayed samples 1 and 2.

Figure 8 displays the peaks of various phases of austenite and martensite for NiTi powder and both samples 1–2. NiTi powder shows the presence of austenite (64 Wt. %) and martensite (36 Wt. %) [19]. However, there is a deviation in the phases out of austenite and martensite from equilibrium towards intermetallic phases in both plasma-sprayed NiTi coating layers. According to the Ni-Ti phase diagram, the melting point of NiTi is 1310 °C, at which 50% of Ni or Ti is present in the NiTi phase [12]. When the temperature shifts below 984 °C, NiTi is combined with other phases to create NiTi_2_. The temperature causes NiTi particles to melt, which, in turn, causes reactions and interactions that lead to the creation of different phases. This corresponds to the intermetallic phases present in both coating layers in samples 1 and 2, with some porosity. The intermetallic phases are found mostly in the region of intersplat areas on the connection boundary between the layers. However, the variable parameter of different feed rates controls the compactness and generates porosity along the coating layers. The influence of intermetallic phases on the major phases of austenite and martensite is determined by the thermal response of the samples. This finding leads to determining the transformation temperature of phases during cooling and heating cycles.

### 3.4. Thermal Characterization of the Samples

**Figure 9:**
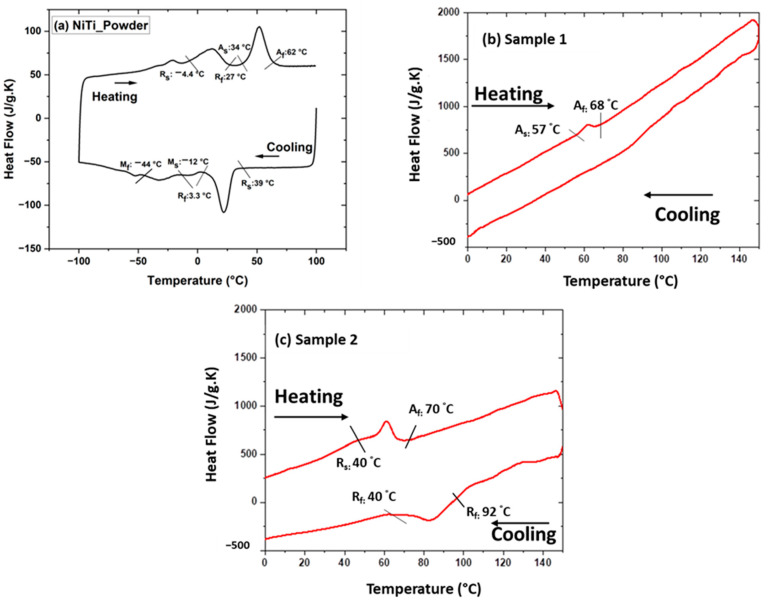
DSC graph of (**a**) NiTi powder; (**b**) sample 1; and (**c**) sample 2 showing transformation temperature during the cooling and heating cycle. NiTi powder shows austenite, R-Phase, and martensite phases; however, the martensite phase is missing in plasma-sprayed samples 1 and 2.

Figure 9a–c displays the transformation temperatures of the powder and coating samples. The powder shows the presence of austenite, R-phase, and martensite during cooling and heating cycles. The formation of the R-phase initiates at 39 °C and ends at 3.3 °C, which leads to the formation of the martensite phase at −12 °C and ends at −44 °C during the cooling cycle in NiTi powder. The powder exhibits the martensite phase below −44 °C. However, upon heating, the R-phase starts at a temperature of −4.4 °C, which proceeds toward the final temperature of 27 °C. Further, the austenite phase starts at 34 °C and ends at 62 °C. Figure 9a shows the NiTi powder in the austenite phase after 62 °C during the heating cycle (Figure 9a). However, in the case of the plasma-sprayed samples, the austenite start and finish temperatures shift towards higher temperatures. Sample 1 (Figure 9b) demonstrated the fact that the austenite phase begins at 57 °C and ends at 68 °C, without clear R-phase and martensite peaks during the cooling cycle. The narrow range of transformation temperatures may lead to narrow hysteresis in the shape memory effect. However, sample 2 ( Figure 9c) shows that the austenite start temperature is 40 °C with a final temperature of 70 °C in the heating cycle, indicating that the R-phase start temperature is 92 °C and ends at 40 °C. These findings are considered to show the temperature window for a thermo-mechanical cycle under a constant load in the cooling and heating cycle.

### 3.5. Thermo-Mechanical Characterization of Samples

**Figure 10:**
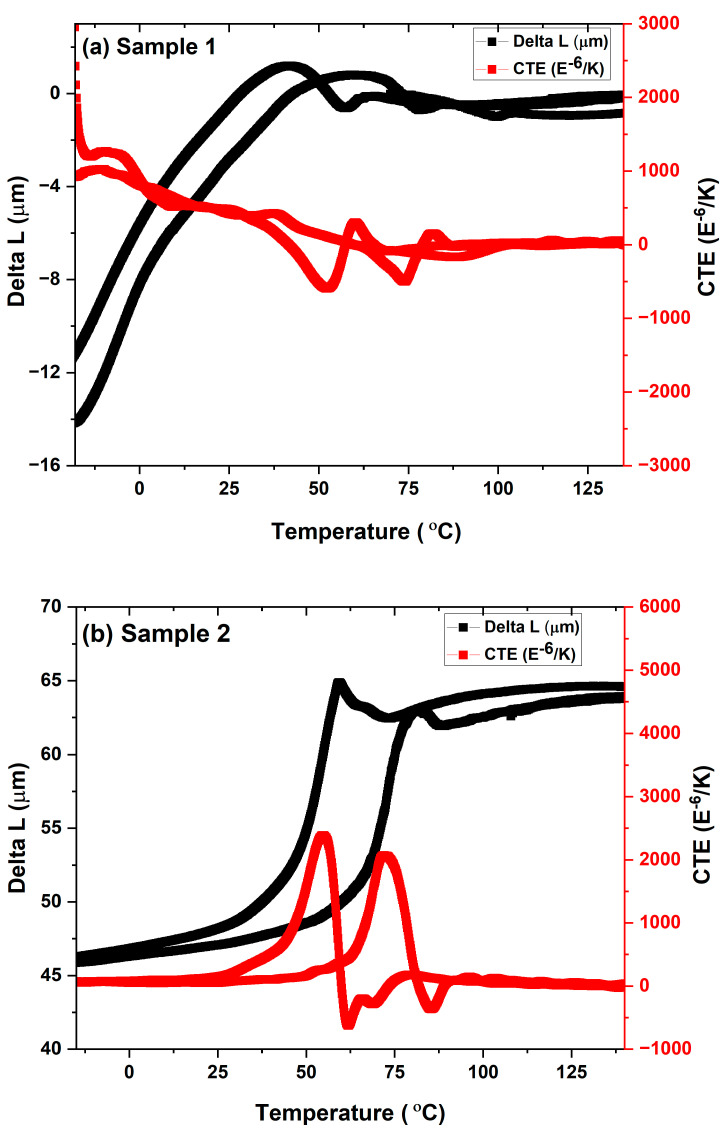
Thermo-mechanical analysis of sample 1 and 2. Delta L and coefficient of thermal expansion (CTE) as a function of temperature.

Figure 10 presents the thermo-mechanical characterization of both samples 1 and 2. Sample 1 exhibits a narrow hysteresis region that arises from the narrow transformation temperature. The very irregular region of hysteresis could be caused by the presence of intermetallic phases, such as pores and segmented crack lines, and by the non-uniform composition of the coating layers. However, sample 2 exhibits a broad hysteresis curve, which could be caused by the phases’ wide range of transformation temperatures. Both samples show recovery with displacement on the heating cycle, which signifies the shape memory behavior. The return of the sample may not coincide well with the same position, indicating the accumulation of residual stress during the thermal cycles. The transformation temperature in samples 1 and 2 is consistent with the phase’s start and finish temperatures, as determined by using the coefficient of thermal expansion (CTE). The first peak corresponds to the start R_f_ temperature during the cooling cycle (R_f_: 40 °C), and the second one corresponds to the final austenite transformation temperature (Af: 70 °C) during the heating cycle for sample 2.

## 4. Discussion

**Figure 11:**
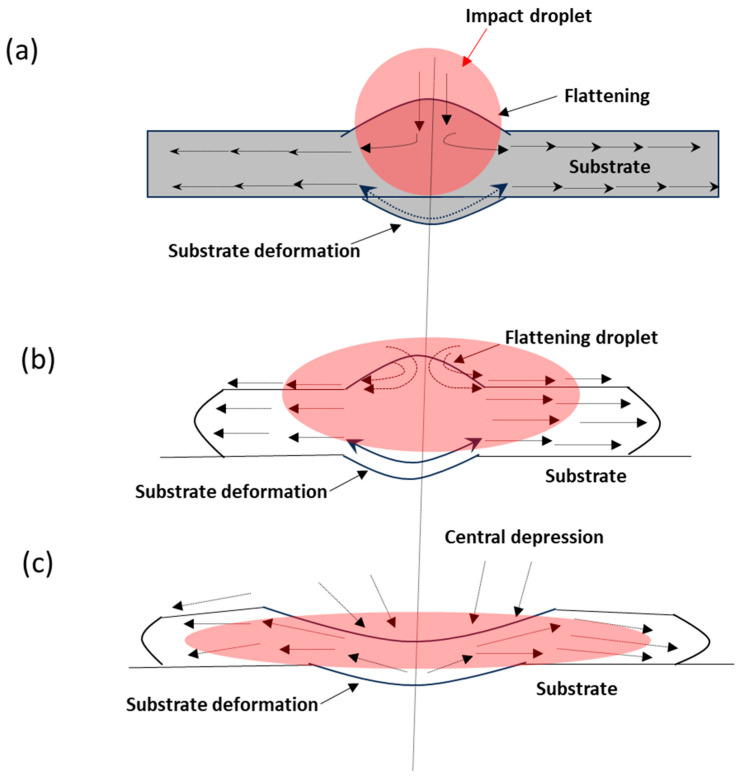
Schematic diagram of molten NiTi particles impacting the stainless steel substrate, showing flattening and spreading behavior. (**a**) Initial stage of contact of a molten particle on the surface of the substrate; (**b**) spreading of the splat; (**c**) droplet splashing with central depression.

Temperature has a significant role in the melting of the particles and the cooling of the substrate in the spraying process. Molten particles begin to spread on the substrate’s surface. Preheating promotes the spreading of particles on the surface of the substrate in more significant ways. Figure 11a–c presents the spreading of molten particles on the substrate surface from initial contact to the final position following the second stage of spreading and then, finally, the splashing of the droplet within the central depression region. Upon impact of a droplet on the substrate surface, a localized mechanical deformation is produced that promotes interlocking adherence of splats to the surface. The surface viscosity of the molten particles promotes spreading on the surface (Figure 11b). There is a little depression in the impact area of the substrate surface (Figure 11c). Nevertheless, in this experiment, spreading along the substrate’s surface can result in undetectable micro-deformation in the nearby area. The preheating of the substrate helps ensure less difference in temperature from the melting of the droplet toward the deposition temperature. As a result, there is less chance of phase deviation as a function of temperature, which was predicted from the phase diagram of Ni-Ti. The deposition process is composed of several stages, from initial melting to final deposition. Thermal plasma arc initiates the melting of particles that reach the arc zone, which begins the first stage of the reaction process. In the second stage, melted particles accelerate and impact the surface of the substrate. The third stage displays the spreading of splats on the surface.

**Figure 12:**
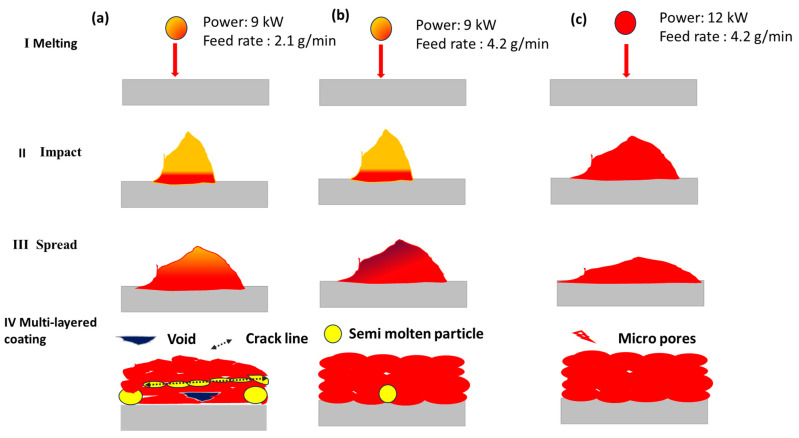
Various stages (**a**–**c**) of particles from melting to impact, with spreading and final formation of the coating layers.

Figure 12 shows the various stages of the deposition process during the melting of the particles in the plasma arc. When plasma gases such as Ar + H_2_ create an arc at a power of 9 kW, the charge explodes at high temperatures. The powder feeder injects the particles at a feed rate of 2.1 g/min or 4.2 g/min into the plasma arc. When the powder enters a high-temperature plasma arc zone, it begins to melt quickly, converting to droplets. The droplets travel with high velocity from the plasma zone toward the substrate. The molten droplets splash upon the substrate with impact force. The impact force causes the particles to disperse laterally, and the substrate temperature causes them to solidify quickly, forming a single splat. Various splats deposited on the substrate form a coating layer (Figure 12a). However, the degree of the melting of the particles is different under different plasma powers, which results in a significant difference in the microstructure and mechanical properties of the coating layers. According to the experiment results, it can be considered that the deposition behavior is based on three categories. At a lower power of 9 kW, the particles are close to the semi-molten mode. The particles that pass through the outer zone of the plasma arc form a partially molten splat in the coating layers. The partially molten splat creates voids within the coating layers that generate porosity. When the feed rate increased from 2.1 to 4.2 g/min at a power of 9 kW, the porosity was reduced. The compact structure of the coating could be formed at a higher feeding rate with higher input power (Figure 12c).

**Figure 13:**
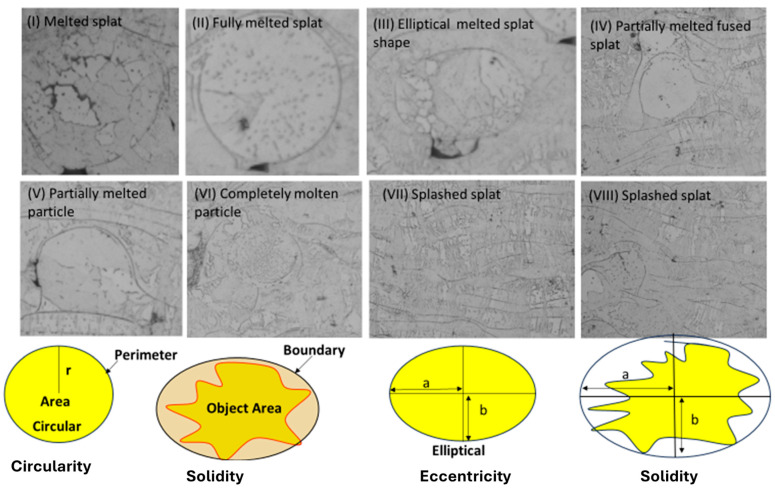
Various morphologies of melted droplets of spraying and evolution of the corresponding melted splat of various areas, where b and a represent the axis in elliptical and partially molten splats.

When the particle melts in the plasma arc during spraying, it creates partially molten splats with circular and elliptical shapes in the coating layers. Figure 13 shows the various forms of splat forms produced in the plasma spraying process during the deposition of the coating layers. A specific number of splats are considered in the coating layers, with partially and completely splashed molten splats produced during the deposition process. The melting parameters depend upon the particle size and position in the plasma arc during flow and deposition on the substrate. The eccentric shape of the melted particle varies from minimum to optimum forms of circular shape. On the other hand, the circular shape shifts away from being perfectly round to being just slightly less round than normal. In the case of solidity, the case shifted from circular to eccentric.

As a result, the circularity, eccentricity, and solidity of the shape are determined by using the following equations [32]:

(1) $$\mathrm{Circularity}=\frac{4\pi \mathrm{Area}}{{\mathrm{Perimeter}}^{2}}$$

(2) $$\mathrm{Eccentricity}=\sqrt{1-\frac{b^{2}}{a^{2}}}$$

(3) $$\mathrm{Solidity}=\frac{\mathrm{object}\;\mathrm{area}}{\mathrm{Bounding}\;\mathrm{area}}$$

**Figure 14:**
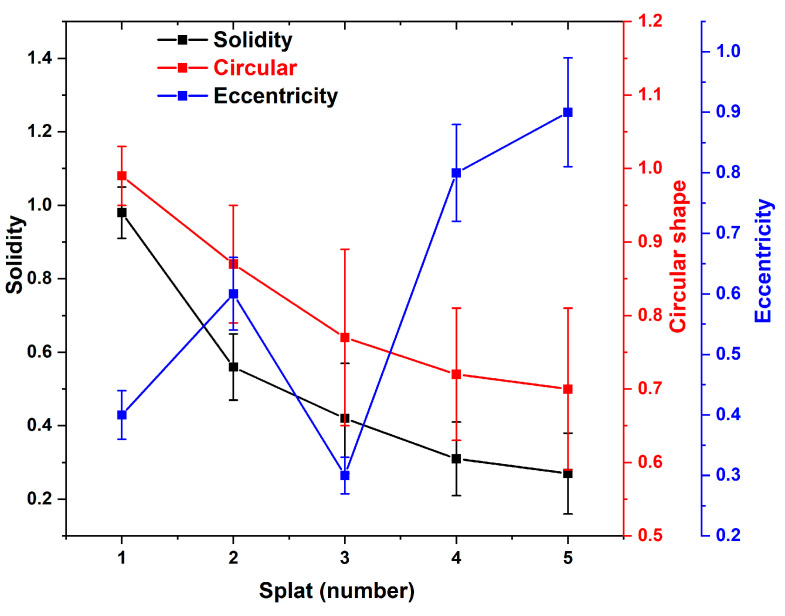
Splats with various areas from circular, elliptical to solid areas in the spraying process.

Utilizing Equations (1)–(3), the various splats and their correlations with the circular, eccentric, and solid areas are calculated and plotted in Figure 14. The uncertainties vary in terms of the number of splats taken into consideration.

The number of splats has an inverse relationship with the solidity and circularity of the splat shape. When the splat shape is close to circularity, the uncertainty error value is lower; when the splat shape deviates from circularity, the uncertainty error value is larger. Solidity is calculated using the object area to the bounding area ratio, which produces a uniform uncertainty error number for all splats that are considered. The most significant divergence in uncertainty error could result from the different elongated forms of the sprayed splat that developed during cooling.

## 5. Conclusions

Multilayer NiTi coatings were prepared using the thermal plasma spraying process using different feed rates. The plasma spraying method was implemented with two variable feed rates that influenced the compactness of the coating layer and determined the pores in the structure. Feedstock NiTi powders with a feed rate of 4.2 g/min create a dense coating layer without any crack line. Both plasma-sprayed samples show higher transformation temperatures for phases during the cooling and heating thermal cycle. This shifting toward higher temperature may be attributed to the intermetallic compounds such as NiTi_2_, which was confirmed in the phase analysis. The shape memory effect of the plasma-sprayed samples exhibits a narrow region of response that may arise from the coating layers with porosity and cracks. However, sample 2 shows a better hysteresis region with shape memory effect with corresponding transformation temperature. The dense layer of the coating prevents any diffusion of foreign elements or impurities into the system. This coating without porosity shows a shape memory effect with a similar response to NiTi shape memory alloys. This process could open the possibilities for using material surface protection using a functional coating of NiTi. Future studies will focus on the impact resistance, scratch, and wear behavior of the coating to emphasize the shape memory effect of NiTi alloy.

## Figures and Tables

**Table 1:** Spraying parameter for the multilayered coating for both samples (1 and 2).

Sample	Substrate	Plasma Power (kW)	Powder Feed Rate (g/min)	Moving Speed (mm/s)	Microhardness on Cross-Section (HV)
1	Stainless steel	9	2.1	1	257.4
2			4.2		254.8

Ar (sheath gas, 35, Central, 10) + H_2_ (1.0); carrier gas Ar, 8; preheat: 60 s; spraying plan: 60 × 6; net powder spray time: 360 s.

## Data Availability

The data is confidential due to the project policy. However it will be available on reader request.
